# Rectus sheath hematoma associated with commencement of therapeutic low molecular weight heparin injections: a case report

**DOI:** 10.1186/s13256-022-03318-6

**Published:** 2022-03-01

**Authors:** Yuxuan Zhou, Kingsley Logan

**Affiliations:** 1 Department of General Medicine, Whangarei Hospital, Northland District Health Board, Whangarei, New Zealand; 2 Whangarei Hospital, Private Bag 9742, Whangarei, 0148 New Zealand

**Keywords:** Low molecular weight heparin, Rectus sheath hematoma, Complication, Acute abdomen, Inferior epigastric artery

## Abstract

**Background:**

Subcutaneous low molecular weight heparin is a commonly used anticoagulant. Catastrophic hemorrhage is a known adverse outcome associated with anticoagulant use. Of all potential bleeding sites, hemorrhage into the rectus sheath is a rare and unusual complication. In this case report, we document a patient who developed rectus sheath hematoma following new commencement of therapeutic low molecular weight heparin.

**Case presentation:**

A 71-year-old New Zealand European woman presented to a peripheral hospital with suspected unstable angina. She was started on therapeutic subcutaneous low molecular weight heparin. While awaiting inpatient transfer to a tertiary hospital for coronary angiography, she developed a large rectus sheath hematoma associated with hemodynamic instability. She required an urgent laparotomy to decompress the hematoma and achieve hemostasis. Postoperatively, her anticoagulation therapy was stopped, and she made a full recovery.

**Conclusion:**

Rectus sheath hematoma is a condition that is difficult to diagnose. The risk of adverse events must always be considered against the indication and potential benefits of new medications, especially with high-risk medications such as anticoagulants.

## Introduction

Subcutaneously administered low molecular weight heparin (LMWH) is a common medication used to treat a wide range of thrombotic conditions. As with all medications, LMWH use can be associated with risks and adverse events. In this case report, we describe rectus sheath hematoma (RSH) as a rare adverse event following the new commencement of therapeutic LMWH. We aim to highlight the clinical presentation, diagnosis, management, and relevant anatomy of RSH in this report.

## Case report

A 71-year-old healthy and independent New Zealand European female presented to a peripheral hospital with new onset central chest pain. Her electrocardiogram (ECG) showed normal sinus rhythm without ischemic changes, and her serial troponin blood tests were unremarkable. Given that her age was a risk factor for cardiac events, she was admitted to the general medicine ward for suspected unstable angina. She was commenced on subcutaneous therapeutic LMWH at a dose of 80 mg twice daily while awaiting transfer to a tertiary hospital for coronary angiography.

On day 3 of her admission, while still awaiting transfer, the patient noticed new cough, worsening lower abdominal pain, and an enlarging suprapubic mass. A bladder scan was performed showing a postvoid residual volume of 87 mL. The mass was thought to be a palpable bladder when assessed by a junior doctor, and an indwelling catheter (IDC) was inserted. All vital observations were within acceptable limits, including her blood pressure remaining stable at 148/91 mmHg. Her hemoglobin from earlier in the day was 131 g/L.

**Fig. 1:**
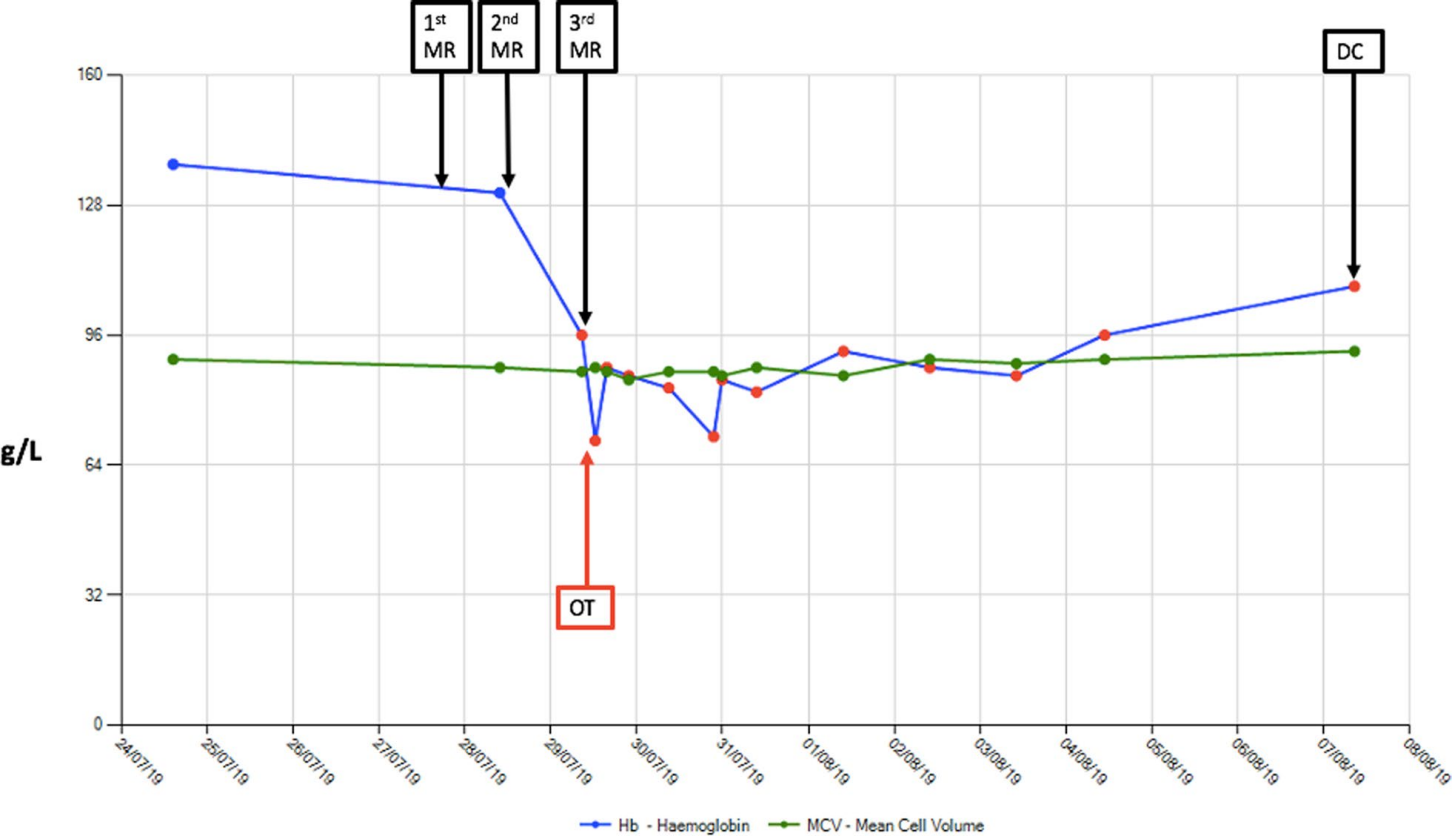
Cumulative trends of hemoglobin g/L (blue) and mean cell volume fL (green) during the patient’s admission. *MR* medical review, *OT* operating theater, *DC* discharge

**Fig. 2:**
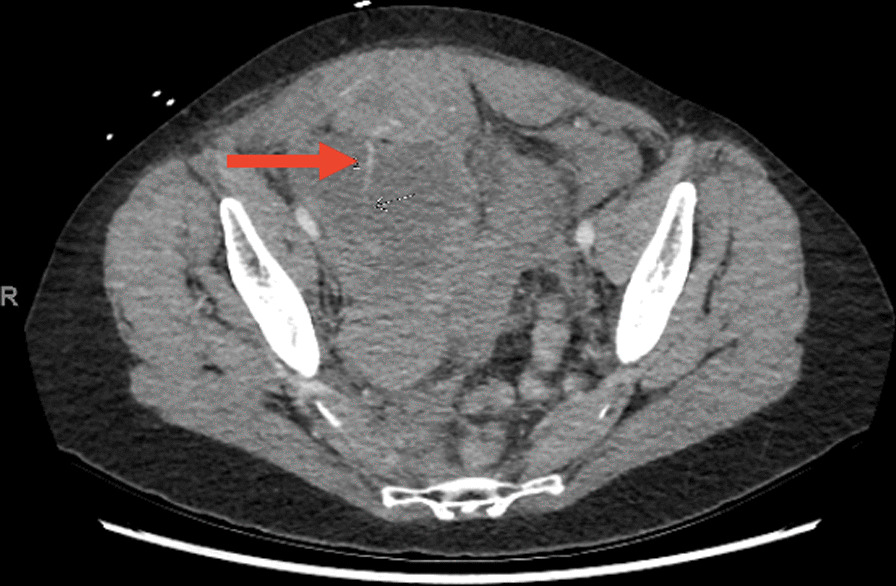
Axial slice of arterial phase contrast computed tomography abdominal scan showing a jet of active arterial bleeding (red arrow) from the right inferior epigastric artery

The next morning (day 4 of her admission), her pain had worsened, and her blood pressure fell acutely to 80/40 mmHg. The mass was still palpable despite her IDC remaining *in situ*. A total of 250 mL drained through her IDC overnight. Repeat physical examination showed a hard tender mass in the suprapubic area of approximately 15 cm diameter. Localized peritonism was present, with normal bowel sounds. An urgent repeat hemoglobin test was performed, which showed a drop to 84 g/L (Fig. 1). An urgent contrast computed tomography (CT) scan was also performed, revealing a large RSH with an active jet of arterial bleeding from the right inferior epigastric artery (IEA) (Fig. 2).

The patient underwent an emergency laparotomy to decompress the hematoma and achieve hemostasis. Intraoperatively, no active bleeding was seen from any major blood vessels. However, generalized venous ooze was present throughout the rectus abdominis muscle. At the end of the procedure, the right IEA was sutured and ligated.

Postoperatively, all anticoagulation therapy was withheld. A CT aortogram (study indication unrelated to her unstable angina presentation) from 2 months prior was reviewed by an inpatient cardiologist who felt that there was unlikely to be ischemia of her coronary vessels. She remained well on the ward and was discharged on day 12 of her admission. Ultimately, she did not require transfer to a tertiary hospital for coronary angiography, and her diagnosis was considered “noncardiac chest pain.”

For completeness, she was followed up in a cardiology outpatient clinic 3 months after discharge. She had remained symptom free with regard to chest and abdominal pain. Her troponin remained normal at follow-up. Outpatient echocardiogram and ECG tracings were all unremarkable. Subsequent full hemostasis screening, including platelet function and clotting factor assays, were all within normal limits, and the patient was discharged from cardiology care.

## Discussion

RSH associated with LMWH is uncommon, with only single case reports or short case series documenting this complication in the literature [1–5]. Most of these patients have been anticoagulated for prolonged periods of time or were medically comorbid [6, 7].

By convention, LMWH is generally administered subcutaneously under the skin around the anterior abdominal wall. It is tempting to attribute this complication to puncture of the IEA by the accidental intramuscular administration of LMWH. However, the needles used are very short (less than 1 cm) and struggle to penetrate deep enough to reach the IEAs. More plausibly, this complication occurred from a combination of the local effects of LMWH in conjunction with cough or strain of the rectus muscles leading to shear injury of the IEA. In a limited case series by Tsapatsaris *et al.*, three of the four patients described developed RSH in association with cough and respiratory infection [8].

The signs and symptoms of RSH can mimic intraabdominal pathology. As a result, RSH is a very difficult condition to diagnose. Maharaj *et al.* described a modified Carnett’s test used to distinguish pain arising from the abdominal wall versus intraabdominal pathology [9]. With the patient sitting halfway up, contraction of their rectus muscles leads to (1) increased tenderness of the mass and (2) the mass being more difficult to palpate. The presence of both signs indicates a positive finding for the test. The increased tenderness upon contraction is a result of compression of the hematoma, while the mass becomes more difficult to palpate because the anterior rectus sheath is taut upon contraction.

Anatomically, a hematoma confined within the rectus sheath cannot extend beyond the *linea alba*. This anatomic consideration is most relevant when using ultrasound to diagnose the condition. However, contrast CT scans are more reliable when diagnosing RSH [10]. Although not reported in the literature, there is the anatomical exception of a posterior RSH extending inferiorly beyond the arcuate line and therefore crossing the midline of the abdomen. From another anatomical perspective, RSH associated with intraabdominal hemorrhage is more suggestive of penetrating iatrogenic injury, as the transversalis fascia and peritoneum itself must be breached for this to occur.

## Conclusion

In summary, LMWH-associated RSH is a rare complication that can be challenging to diagnose. However, missed diagnosis can lead to severe and even fatal consequences. A broad differential diagnoses list in conjunction with detailed history taking, clinical examination, and appropriate investigations can result in the correct diagnosis being made promptly. To minimize the risk of this complication occurring, care must be taken to consider the indication, risks, and benefits before starting any new medication.

## Abbreviations

CT: Computed tomography
ECG: Electrocardiogram
IDC: Indwelling catheter
IEA: Inferior epigastric artery
LMWH: Low molecular weight heparin
RSH: Rectus sheath hematoma
cm: Centimeters
mL: Milliliters
mmHg: Millimeters of mercury
L: Liters
g: Grams

## Declarations

### Availability of data and materials

Not available due to privacy policy.

## References

[1] Donaldson J, Knowles CH, Clark SK, Renfrew I, Lobo MD. Rectus sheath haematoma associated with low molecular weight heparin: a case series. Ann R Coll Surg Engl. 2007;89(3):309–12. 10.1308/003588407X179152 PMC1964713 17394721

[2] Kayrak M, Bacaksiz A, Yazici M. Is enoxaparin injection from the abdominal wall safe in elderly people?: a fatal case of rectus sheath hematoma. Can Fam Physician. 2008;54(9):1246–8. PMC2553457 18791099

[3] Sullivan LEJ, Wortham DC, Litton KM. Rectus sheath hematoma with low molecular weight heparin administration: a case series. BMC Res Notes. 2014;7:586. 10.1186/1756-0500-7-586 PMC4162921 25178308

[4] Ortega-Carnicer J, Ceres F. Rectus sheath haematoma with severe haemodynamic compromise after enoxaparin use for unstable angina. Resuscitation. 2003;57(1):113–4. 10.1016/s0300-9572(02)00431-8 12668308

[5] Fitzgerald JEF, Fitzgerald LA, Anderson FE, Acheson AG. The changing nature of rectus sheath haematoma: case series and literature review. Int J Surg. 2009;7(2):150–4. 10.1016/j.ijsu.2009.01.007 19261556

[6] Cherry WB, Mueller PS. Rectus sheath hematoma: review of 126 cases at a single institution. Medicine. 2006;85(2):105–10. 10.1097/01.md.0000216818.13067.5a 16609349

[7] James RF. Rectus sheath haematoma. Lancet. 2005;365(9473):1824. 10.1016/S0140-6736(05)66587-7 15910957

[8] Tsapatsaris NP. Low-dose heparin: a cause of hematoma of rectus abdominis. Arch Intern Med. 1991;151(3):597–9. 10.1001/archinte.151.3.597 1845603

[9] Maharaj D, Ramdass M, Teelucksingh S, Perry A, Naraynsingh V. Rectus sheath haematoma: a new set of diagnostic features. Postgrad Med J. 2002;78(926):755–6. 10.1136/pmj.78.926.755 PMC1757949 12509696

[10] Berná JD, Garcia-Medina V, Guirao J, Garcia-Medina J. Rectus sheath hematoma: diagnostic classification by CT. Abdom Imaging. 1996;21(1):62–4. 10.1007/s002619900011 8672975

